# Immediate and sustained increases in the activity of vagal preganglionic neurons during exercise and after exercise training

**DOI:** 10.1093/cvr/cvad115

**Published:** 2023-07-30

**Authors:** Alla Korsak, Daniel O Kellett, Qadeer Aziz, Cali Anderson, Alicia D’Souza, Andrew Tinker, Gareth L Ackland, Alexander V Gourine

**Affiliations:** Centre for Cardiovascular and Metabolic Neuroscience, Department of Neuroscience, Physiology and Pharmacology, University College London, Gower Street, London WC1E 6BT, UK; Centre for Cardiovascular and Metabolic Neuroscience, Department of Neuroscience, Physiology and Pharmacology, University College London, Gower Street, London WC1E 6BT, UK; Centre for Cardiovascular and Metabolic Neuroscience, Department of Neuroscience, Physiology and Pharmacology, University College London, Gower Street, London WC1E 6BT, UK; Centre for Clinical Pharmacology and Precision Medicine, William Harvey Research Institute, Queen Mary University of London, London, UK; Division of Cardiovascular Sciences, University of Manchester, Manchester, UK; Division of Cardiovascular Sciences, University of Manchester, Manchester, UK; Centre for Clinical Pharmacology and Precision Medicine, William Harvey Research Institute, Queen Mary University of London, London, UK; Translational Medicine and Therapeutics, William Harvey Research Institute, Queen Mary University of London, London, UK; Centre for Cardiovascular and Metabolic Neuroscience, Department of Neuroscience, Physiology and Pharmacology, University College London, Gower Street, London WC1E 6BT, UK

**Keywords:** Brain, Exercise, Heart, Parasympathetic, Vagus

## Abstract

**Aims:**

The brain controls the heart by dynamic recruitment and withdrawal of cardiac parasympathetic (vagal) and sympathetic activity. Autonomic control is essential for the development of cardiovascular responses during exercise, however, the patterns of changes in the activity of the two autonomic limbs, and their functional interactions in orchestrating physiological responses during exercise, are not fully understood. The aim of this study was to characterize changes in vagal parasympathetic drive in response to exercise and exercise training by directly recording the electrical activity of vagal preganglionic neurons in experimental animals (rats).

**Methods and results:**

Single unit recordings were made using carbon-fibre microelectrodes from the populations of vagal preganglionic neurons of the nucleus ambiguus (NA) and the dorsal vagal motor nucleus of the brainstem. It was found that (i) vagal preganglionic neurons of the NA and the dorsal vagal motor nucleus are strongly activated during bouts of acute exercise, and (ii) exercise training markedly increases the resting activity of both populations of vagal preganglionic neurons and augments the excitatory responses of NA neurons during exercise.

**Conclusions:**

These data show that central vagal drive increases during exercise and provide the first direct neurophysiological evidence that exercise training increases vagal tone. The data argue against the notion of exercise-induced central vagal withdrawal during exercise. We propose that robust increases in the activity of vagal preganglionic neurons during bouts of exercise underlie activity-dependent plasticity, leading to higher resting vagal tone that confers multiple health benefits associated with regular exercise.

**Translational perspective**

The data obtained in this study contribute to our understanding of the fundamental mechanisms underlying the control of the heart by the autonomic nervous system. As we grow older and develop cardiometabolic diseases (e.g. hypertension, diabetes mellitus), there is a progressive decline in cardiac vagal activity. Loss of vagal activity impairs the ability of the heart to rapidly respond to changes in behavioural/physical demands, limiting our ability to exercise. This research suggests that the development of novel therapeutic approaches designed to recruit vagal activity (for example by autonomic neuromodulation) may improve exercise tolerance, increase quality of life, and promote cardiovascular health.

**Time of primary review: 40 days**

## 1. Introduction

Physical activity is essential for every aspect of physiological, emotional, and cognitive health. Regular exercise provides multiple health benefits, and higher exercise capacity is strongly associated with reduced risk of cardiovascular disease, type 2 diabetes, malignancy, osteoporosis, depression, and premature death.^1–3^ The ability to mount a strong cardiovascular response during exercise (essential to meet the metabolic demands of working muscles, while maintaining systemic blood pressure and perfusion of all the organs) is a prerequisite for achieving higher exercise performance. Yet, the physiological control mechanisms responsible for orchestrating cardiac responses during exercise are not fully understood.

The autonomic nervous system controls the heart by dynamic recruitment and withdrawal of cardiac parasympathetic (vagal) and sympathetic activities. A prevailing model of autonomic control of the heart during exercise has long held that increases in heart rate (HR) from resting baseline initially occur through (nearly complete) vagal withdrawal, followed by gradual activation of the sympathetic input.^4–6^ It has been suggested that rapid feedback from muscle mechanoreceptors at the initiation of exercise contributes to the withdrawal of cardiac vagal activity.^5^ Another commonly accepted view is that exercise training increases vagal tone and that this increase is largely responsible for low resting heart rates recorded in trained individuals and especially in endurance athletes.^6^

However, changes in parasympathetic vagal activity during exercise and in response to exercise training have never been directly assessed. The concepts of rapid vagal withdrawal during exercise and of increased resting vagal activity as a result of exercise training are based on data from experimental studies which used pharmacological autonomic blockade, and/or indirect measures of cardiac autonomic function, such as heart rate variability (HRV) analysis.^5^ However, the interpretation of HRV as a measure of autonomic activity is complicated by its strong dependency on respiratory frequency/depth and resting HR.^7^ Moreover, direct recordings of action potential firing within the cervical vagus nerve in experimental animals shows no correlation between vagal activity and any of the common HRV metrics.^8^

The idea of near-complete cardiac vagal withdrawal appears to be at odds with the critical requirement of vagal activity for baroreflex control of the heart in all conditions.^9–12^ Indeed, physiological modelling and experimental studies involving autonomic blockade in healthy volunteers suggested that vagal activity may be maintained during exercise, and even at high exercise loads, vagal inputs may continue to modulate heart function alongside heightened sympathetic activity.^4^

**Figure 1:**
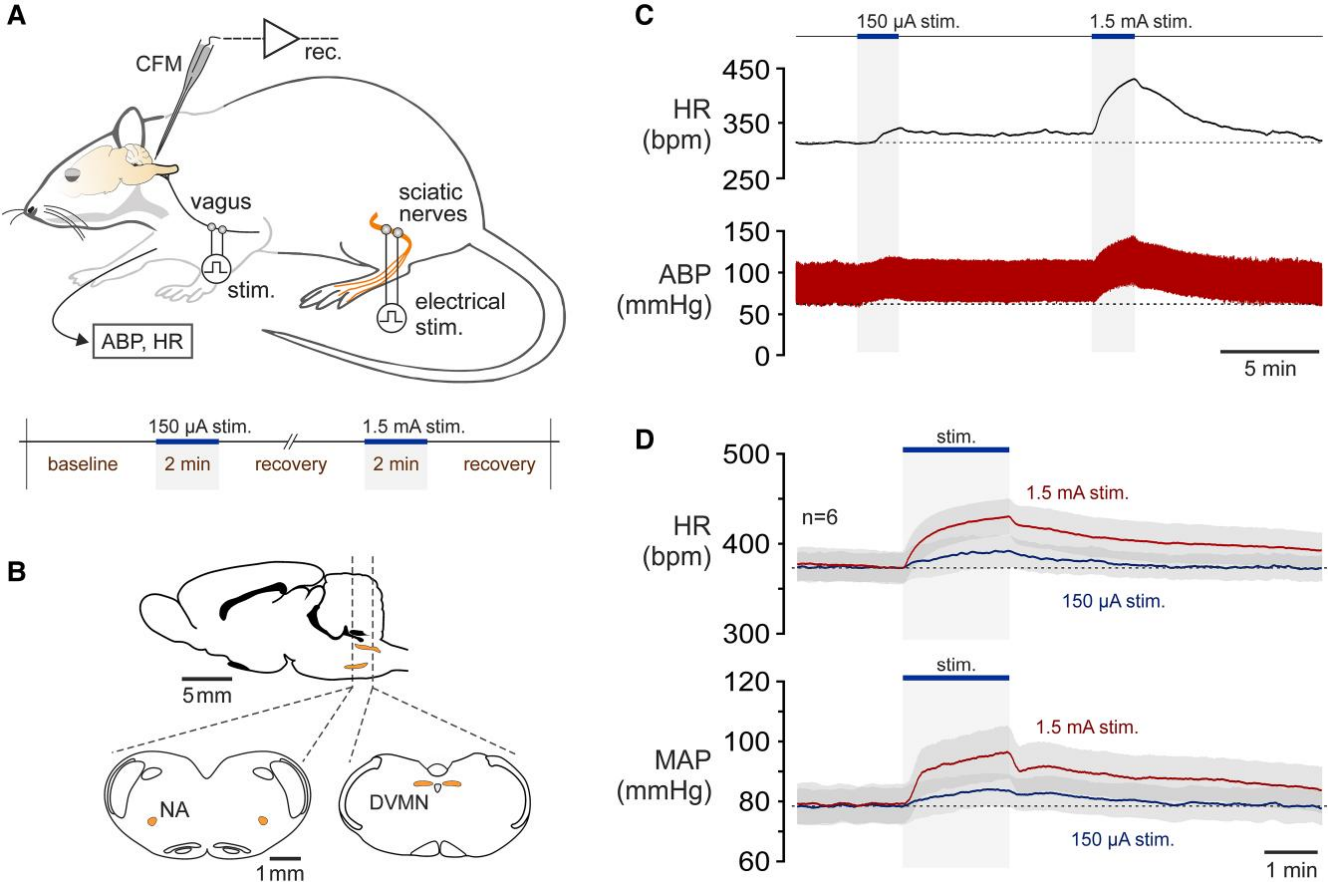
Experimental model to study the activity of vagal preganglionic neurons during exercise. (*A*) A diagram of the experimental setup in anaesthetized rats instrumented for the recordings of the electrical activity (firing of action potentials) of vagal preganglionic neurons in the brainstem using carbon fibre microelectrodes (CFM). The brachial artery was cannulated for the recordings of ABP and HR. Vagal preganglionic neurons were identified by antidromic activation in response to the electrical stimulation of the vagus nerve. The model of exercise used in this study involved pulses of electrical stimulation applied at a frequency of 1 Hz to both sciatic nerves to produce rhythmic muscular contractions, increase plasma lactate concentration, and mimic feedback from the muscle mechanoreceptors. Stimulations were applied for 2 min periods sequentially with currents of 150 µA and 1.5 mA to mimic bouts of ‘low’ and ‘high’ intensity exercise; (*B*) Schematic drawings of the rat brain in parasagittal and coronal projections illustrating the anatomical locations of the populations of vagal preganglionic neurons of the NA and the DVMN; (*C*) Representative raw traces, and (*D*) averaged (means ± s.e.m.) recordings obtained in 6 animals illustrating changes in HR, ABP and mean arterial blood pressure (MAP) induced by low (150 µA) and high (1.5 mA) intensity stimulations of the sciatic nerves.

In this study, we aimed to determine the profile of changes in vagal activity during exercise by directly recording vagal preganglionic neuron firing using carbon-fibre microelectrodes in experimental animals. Recordings were made from the populations of vagal preganglionic neurons residing in the nucleus ambiguus (NA) and the dorsal vagal motor nucleus (DVMN) of the brainstem in rats (*Figure 1A, B*). These two neuronal populations have distinct patterns of discharge and provide differential control of cardiac function.^13,14^ Neurons of the NA receive modulatory inputs from the neighbouring respiratory network and primarily control the nodal tissue and, therefore, HR.^15,16^ Vagal preganglionic neurons residing in the DVMN display a tonic pattern of discharge and provide vagal modulation of ventricular excitability and contractility.^17–19^ Exercise-induced changes in the activity of the cardiac branch of the vagus nerve were also assessed.

We show that the activity of vagal preganglionic neurons in the NA and the DVMN and the activity of the cardiac vagus (CV) increase during bouts of acute exercise. In exercise-trained rats, the resting activity of NA and DVMN neurons was found to be significantly higher compared to that recorded in sedentary animals. These data provide the first direct neurophysiological evidence that exercise training increases vagal tone and argue against the notion of central vagal withdrawal during exercise.

## 2. Methods

All the experiments were performed in accordance with the European Commission Directive 2010/63/EU (European Convention for the Protection of Vertebrate Animals used for Experimental and Other Scientific Purposes) and the UK Home Office (Scientific Procedures) Act (1986) with project approval from the University College London Institutional Animal Care and Use Committee. The rats were group-housed and maintained on a 12-h light cycle (lights on 07:00) and had *ad libitum* access to water and food.

### 2.1 Animal preparation

Young adult male and female Sprague–Dawley rats (280–350 g; 2–3 months old; Charles River, UK) were used in this study. The rats were anaesthetized with urethane (induction: 1.3 g /kg, i.p.; maintenance: 10–25 mg /kg /h, i.v.) and instrumented as described in detail previously.^20–23^ Adequate depth of anaesthesia was monitored and confirmed by the stability of arterial blood pressure (ABP) and HR recordings in response to a paw pinch. The brachial artery and vein were cannulated for the measurements of ABP and the administration of anaesthetic, respectively. The animal was intubated and mechanically ventilated with oxygen-supplemented air using a small rodent ventilator (tidal volume ∼1 mL per 100 g of body weight; ∼60 strokes /min). Arterial PO_2_, PCO_2_, and pH were measured regularly (RAPIDLab 248 pH/Blood Gas Analyzer, Siemens, Farnborough, UK) and maintained within physiological ranges (PO_2_ 95–120 mmHg; PCO_2_ 35–45 mmHg and pH 7.35–7.45) by adjusting the tidal volume, ventilator frequency and/or the level of supplemental oxygen. Body temperature was maintained at 37.0 ± 0.5 °C. The animal was placed in a prone position and the head was secured in a stereotaxic frame. An occipital craniotomy was performed, and the cerebellum was partially removed to expose the dorsal surface of the brainstem. The right cervical vagus nerve and both sciatic nerves were dissected, isolated from the surrounding tissues, placed on silver wire stimulating electrodes, and covered with paraffin.

### 2.2 Recordings of the activity of vagal preganglionic neurons

The electrical activity (firing of action potentials) of neurons in the DVMN or NA was recorded using carbon fibre microelectrodes (CFM).^24^ The recorded signal was amplified (×10 000), filtered (80–1500 Hz), and sampled at a rate of 20 kHz. Both the DVMN and the NA neuronal populations were approached by advancing the electrodes from the dorsal surface of the brainstem (*Figure 1A, B*). Vagal preganglionic neurons were identified by antidromic activation in response to pulses of electrical stimulation applied to the cervical vagus nerve, and their locations were mapped using a stereotaxic atlas with coordinates relative to the *calamus scriptorius*. The accuracy of this approach was validated in a series of preliminary trials with microinjections of Pontamine sky blue dye into the regions of the NA and DVMN using the same manipulator and stereotaxic coordinates.

### 2.3 Recordings of the activity of the cardiac branch of the vagus nerve

Recordings of the activity of the cardiac vagal branch were performed in a separate group of animals anaesthetized and instrumented as described above. The animal was placed in a prone position and the head was secured in a stereotaxic frame, but craniotomy was not performed. A lateral approach was used to expose the right cardiac vagal branch. Portions of the second and the third ribs were removed, and the azygos vein was ligated and retracted. The cardiac branch leaving the thoracic vagus was identified, cleared of connective tissue, placed on a bipolar silver electrode, and covered with paraffin. Correct identification of the cardiac branch of the vagus nerve was confirmed by observing strong bradycardia in response to electrical stimulation (pulse duration 1 ms; frequency 10 Hz; intensity 1 mA). The nerve was crushed using fine forceps distally to the electrodes. Nerve activity was amplified (× 20 000), filtered (80–1000 Hz), and sampled at a rate of 10 kHz.

### 2.4 Experimental model of simulated exercise

Single-unit recordings of vagal preganglionic neuron activity in conscious animals are not feasible because of the difficulties in achieving stability of the brainstem (which is required for electrophysiological recordings of this type), and the need to characterize these neurons by antidromic activation in response to electrical stimulation of the vagus nerve.

In this study, we used an established experimental model of simulated exercise in anaesthetized animals.^25–29^ This model involves pulses of electrical stimulation applied at a low frequency to both sciatic nerves to produce rhythmic muscular contractions and mimic feedback from muscle mechanoreceptors, which are thought to be responsible for vagal withdrawal and sympathetic activation during exercise.^5^ Stimulations (pulse duration 10 ms; frequency 1 Hz; intensity 150 µA or 1.5 mA) of the sciatic nerves were applied for 2 min with an alternating left-right pattern:—each leg stimulation being delayed by 0.5 s with respect to the stimulation delivered to the other limb (*Figure 1A, C*), thus mimicking animal locomotion. Plasma lactate concentration was measured from arterial blood samples taken at baseline and at the peak of simulated exercise (Cobas Accutrend Plus Meter, Roche, Welwyn Garden City, UK).

In a separate group of animals, the cardiovascular responses to this form of simulated exercise were evaluated in conditions of systemic muscarinic receptor blockade with atropine (2 mg/kg; i.v.), β-adrenoceptor blockade with atenolol (5 mg/kg; i.v.), or autonomic blockade following administration of both atropine (2 mg/kg; i.v.) and atenolol (5 mg/kg; i.v.).

### 2.5 Voluntary exercise training

To determine the effect of exercise training on the activity of vagal preganglionic neurons, electrophysiological recordings of NA and DVMN neuronal discharge were made in experiments conducted in rats that were given unrestricted access to running wheels in their standard home cages.^30^ By the end of the 6-week period of housing with the wheels, rats were voluntarily running distances of ∼2.5 km per 24 h, as reported in the literature.^31^ An average distance of 2374 ± 615 m was recorded in a representative group of six rats over a period of 24 h. A control group of 10 animals was housed in standard home cages with running wheels installed, but fixed (not rotating). There was no difference in the activity of vagal neurons recorded in this control group vs. a larger group of sedentary animals (housed without fixed wheels); thus, the data obtained in all sedentary rats have been pooled for the analysis.

### 2.6 Optogenetic stimulation of vagal preganglionic neurons

Vagal preganglionic neurons characteristically express the transcriptional factor Phox2 and were targeted to express a light-sensitive chimeric channelrhodopsin derivative ChIEF fused with a fluorescent protein tdTomato (ChIEF-tdTomato) or enhanced green fluorescent protein (eGFP; control) using lentiviral vectors (LVV). Transgene expression was driven under the control of a Phox2-activated promoter—PRSx8.^32^ The specificity of the vectors used and the efficacy of light stimulation of the individual DVMN neurons as well as the whole vagus nerve efferent activity have been described in detail previously.^18,33–35^

Rats (150–200 g; *n* = 16) were anaesthetized with a combination of ketamine (60 mg/kg; i.m.) and medetomidine (250 µg/kg; i.m.). Adequate depth of surgical anaesthesia was maintained and confirmed by the absence of a withdrawal response to a paw pinch. With the head of the animal in a stereotaxic frame, a midline dorsal neck incision was made to expose the dorsal brainstem surface. DVMN neurons were targeted bilaterally with one microinjection on each side delivering viral particles of either LVV-PRSx8-eGFP (*n* = 7 rats) or LVV-PRSx8-ChIEF-tdTomato (*n* = 8 rats). The microinjections (0.5 µL at a rate of 0.1 µL/min) were made at 0.5 mm rostral, 0.6 mm lateral, and 0.8 mm ventral from the *calamus scriptorius*. After the microinjections, the wound was sutured and anaesthesia was reversed with atipamezole (1 mg/kg, i.m.). Postoperatively, the animals received buprenorphine analgesia for 3 days (0.05 mg/kg per day, s.c.). No complications were observed after the surgery and the animals gained weight normally.

After a period of 4 weeks (to allow high and stable transgene expression in the DVMN), the animals were anaesthetized with isoflurane (induction: 5%; maintenance: 2–2.5% in oxygen-enriched air). The femoral artery was cannulated to record ABP. Adequate depth of anaesthesia was monitored by the stability of ABP and HR recordings, which did not show responses to a paw pinch. An occipital craniotomy was performed, and the dorsal surface of the brainstem was exposed. For optical stimulation of the DVMN neurons, laser light (445 nm) was applied to the dorsal brainstem surface via an optrode (Ø 0.2 mm; Art Photonics, Berlin, Germany). Optogenetic stimulation of efferent vagus nerve activity (in animals transduced to express ChIEF-tdTomato) or sham-stimulation (in animals transduced to express eGFP) was applied at a frequency of 5 Hz (10 ms light pulses) for a period of 4 h. After the period of stimulation, the animals were given an overdose of isoflurane, the hearts were removed, the sinoatrial (SA) node was dissected (as the intercaval region adjacent to the crista terminalis and bordered by the atrial septum,^36,37^ frozen in liquid nitrogen and kept at −80°C until assayed. The brains were removed and fixed in phosphate-buffered (0.1 M, pH 7.4) 4% paraformaldehyde solution. After cryoprotection (30% sucrose), the brainstem was isolated, and a sequence of transverse slices (30 µm) was cut to determine the extent of ChIEF-tdTomato or eGFP expression in DVMN neurons.

### 2.7 qPCR analysis of gene expression in the SA node

Total RNA was extracted from the SA node biopsies using an RNeasy Mini kit (Qiagen, Manchester, UK), according to the manufacturer’s instructions. An on-column DNase I treatment (Qiagen) was performed to eliminate genomic DNA contamination. RNA quantity and quality were measured by absorption at 260 and 280 nm wavelengths, using a Nanodrop1000 spectrophotometer. A High-Capacity RNA-to-cDNA™ kit (Applied Biosystems, #4387406) was used for cDNA preparation. qPCR was performed using *Power* SYBR Green PCR Master Mix (ThermoFisher, Loughborough, UK) on a QuantStudio 7 Flex Real-Time PCR system. All reactions were run in duplicate. Amplification plots were observed and manual adjustment of the threshold was performed where required using Connect software (ThermoFisher). The expression of three constitutively active reference genes was assessed in all samples. The Genorm algorithm (Statminer software; Tibco, London, UK) was used to identify the most stably expressed reference gene(s) for data normalization. A combination of *Rn18s* and *Gapdh* was used. Mean Ct values were calculated for all the samples for both the reference genes and the genes of interest. Relative gene expression was calculated using the ΔCt method and transformed (2^−ΔCt^). Expression of the following genes was analysed (Qiagen QuantiTect cat no of the primers are given in parentheses): *Adrb1*: β1-adrenoceptor (QT00429247); *Adrbk2*: G-protein-coupled receptor kinase 2 (GRK2) (QT00184177); *Arrb2*: β-Arrestin-2 (QT00188433); *Cacna1d*: α1d subunit of the L-type Ca^2+^ channel (QT00194306); *Chrm2*: M2 muscarinic receptor (QT00380541); *Egr1*: early growth response 1 (QT02423414); *Fos*: c-Fos (QT01576330); *Hcn4*: hyperpolarization activated cyclic nucleotide gated channel 4 (QT00191387). The reference genes were: *Rn18s* (QT02589300); Gapdh (QT00199633); and *Hprt* (QT00199640).

### 2.8 Data acquisition and analysis

Recordings of neuronal activity and cardiovascular variables were obtained and analysed using *Spike2* software (Cambridge Electronic Design, Cambridge, UK). Statistical analysis of the data was performed using GraphPad Prism software. The normal distribution of data was determined by the Shapiro–Wilk test. Mann–Whitney *U* test, Student’s *t*-test, or analysis of variance (ANOVA) followed by Tukey’s multiple comparison tests were used to compare the paired and un-paired data between the experimental groups, as appropriate. The data are reported as individual values and means ± s.e.m. Differences with a *P* value of less than 0.05 were considered to be significant.

## 3. Results

### 3.1 Characterization of the exercise model

The reflexes originating in exercising muscles are largely responsible for the development of cardiovascular responses during exercise, with the contribution of inputs from higher brain centres (‘central command’) at exercise onset.^5,38^ In this study, we used an experimental model of simulated exercise in anaesthetized animals developed by Waldrop^25–28^ with modifications.^29^ In urethane-anaesthetized rats, left and right sciatic nerves were stimulated at 1 Hz with an alternating left-right pattern (each nerve stimulation being delayed by 0.5 s with respect to stimulations given to the opposite side) to produce rhythmic hindlimb muscular contractions and mimic feedback from muscle mechanoreceptors, thought to be responsible for vagal withdrawal during exercise.^5^ Stimulations were applied for 2 min periods with currents of 150 µA or 1.5 mA to mimic bouts of ‘low’ and ‘high’ intensity exercise (*Figure 1A, C*), leading to the development of stereotypical cardiovascular responses, involving stimulation intensity-dependent simultaneous increases in HR and ABP (so-called exercise-induced baroreceptor re-setting^4^) (*Figure 1C, D*), similar to those occurring during ‘natural’ exercise. In a representative group of 6 animals, mean ABP increased from 78 ± 6 mmHg to 83 ± 5 mmHg (*P* = 0.005) and from 79 ± 7 mmHg to 96 ± 9 mmHg (*P* = 0.003) at the end of the 2-min period of stimulation at 150 µA and 1.5 mA intensity, respectively (*Figure 1D*). HR increased from 373 ± 16 bpm to 392 ± 17 bpm (*P* = 0.031) and from 373 ± 18 bpm to 430 ± 20 bpm (*P* < 0.001) in response to low- and high-intensity stimulation, respectively (*Figure 1D*). In this model of exercise, high-intensity stimulation of the hindlimbs led to a significant increase in plasma concentration of lactate (from 3.3 ± 0.2 mM to 4.4 ± 0.2 mM; *P* < 0.001; *n* = 9).

**Figure 2:**
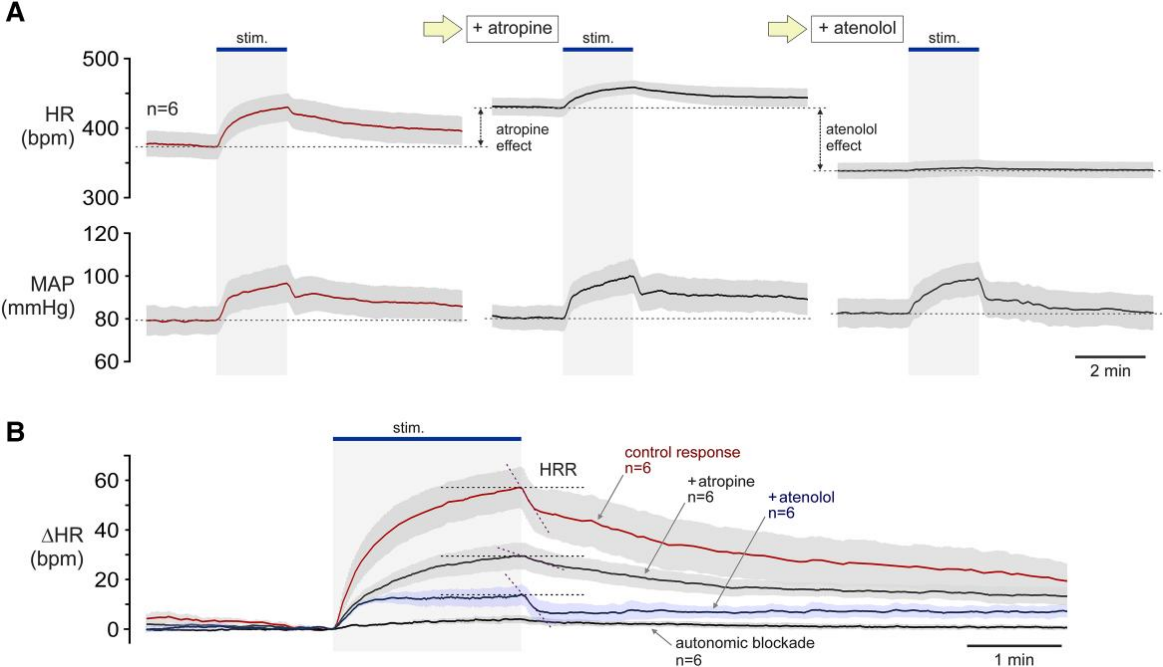
Characterization of the exercise model. (*A*) Averaged traces of the recordings obtained sequentially in six animals illustrating changes in HR and MAP induced by high (1.5 mA) intensity stimulation of the sciatic nerves in control conditions, in conditions of systemic muscarinic receptor blockade with atropine (2 mg/kg; i.v.), and then in conditions of autonomic blockade following the administration of β-adrenoceptor blocker atenolol (5 mg/kg; i.v.); (*B*) Summary data illustrating the relative changes in HR induced by 1.5 mA intensity sciatic nerve stimulation applied in control conditions, after systemic administration of atropine or atenolol, and in conditions of autonomic blockade following combined treatment with atropine and atenolol. The effects of muscarinic and/or β-adrenoceptor blockade on cardiovascular responses to sciatic nerve stimulation in these experiments were found to be similar to those observed in human exercise studies involving autonomic blockade.^40^ HRR, heart rate recovery after exercise (HRR slope is reduced by atropine).

For further characterization of this model, cardiovascular responses to sciatic nerve stimulation-induced muscular contractions were evaluated in conditions of systemic muscarinic receptor blockade with atropine (2 mg/kg; i.v.), β-adrenoceptor blockade with atenolol (5 mg/kg; i.v.), or autonomic blockade following combined treatment with atropine (2 mg/kg; i.v.) and atenolol (5 mg/kg; i.v.) (*Figure 2*). In agreement with previously reported data,^18,39^ we confirmed that vagal and sympathetic influences on the heart are largely preserved under urethane anaesthesia. Administration of atropine increased HR from 377 ± 18 bpm to 431 ± 12 bpm (*n* = 6; *P* = 0.002; *Figure 2A*). Subsequent administration of atenolol reduced HR to 339 ± 11 (*n* = 6; *P* < 0.001; *Figure 2A*).

Under conditions of systemic muscarinic receptor blockade with atropine, absolute peak increases in HR were significantly higher (459 ± 10 bpm; *P* = 0.031) compared to peak HR responses under control conditions (430 ± 20 bpm) (*Figure 2A*), but relative (to baseline) increases in HR were reduced (*Figure 2B*). In response to high-intensity exercise, HR increased by 57 ± 8 bpm at the end of the 2-min period of stimulation. The HR response was reduced by atropine (peak increase 29 ± 5 bpm; *P* = 0.018), atenolol (peak increase 14 ± 4 bpm; *P* < 0.001), and abolished in conditions of autonomic blockade (peak increase 4 ± 2 bpm; *P* < 0.001) (*Figure 2B*). The speed of HR recovery immediately after cessation of the exercise was markedly reduced by atropine and not affected by atenolol (*Figure 2B*), in full agreement with data obtained in human studies.^41^ Systemic treatment with atropine and atenolol had no effect on blood pressure responses to stimulations (*Figure 2A*).

Thus, the effects of muscarinic and/or β-adrenoceptor blockade on cardiovascular responses that develop during periods of muscular contractions induced by sciatic nerve stimulation were found to be very similar to those observed in analogous human exercise studies with autonomic blockade,^40^ confirming the appropriateness of this model to study autonomic control of the heart during exercise.

### 3.2 Exercise increases the activity of vagal preganglionic neurons of the NA

The restraining parasympathetic control of cardiac nodal tissue and, therefore, HR, is provided predominantly by the population of vagal preganglionic neurons of the NA.^14–16^ If central vagal withdrawal contributes to the increases in HR during exercise, then the activity of NA neurons should decrease with increasing workload and rapidly rebound upon cessation of exercise, in line with current models of cardiac autonomic control during exercise.^4–6^

**Figure 3:**
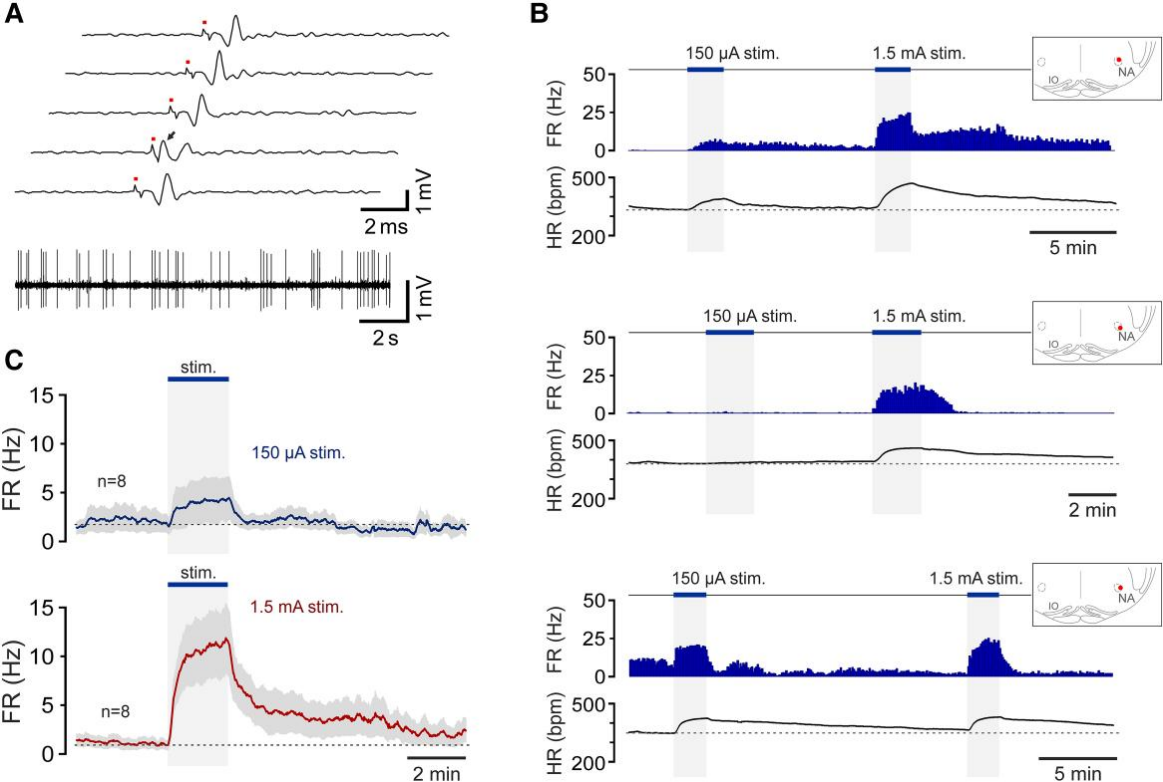
Exercise increases the activity of vagal preganglionic neurons of the NA. (*A*) Identification of vagal neurons of the NA. Five traces illustrate antidromic activation (latency 1.4 ms) of a vagal preganglionic neuron of the NA in response to pulses of electrical stimulation (1.5 mA, 0.2 ms pulses, 1 Hz) applied to the cervical vagus nerve at the time points marked by the dots. On one of the traces, a spontaneous spike (arrow) obscures the antidromic spike. Lower trace illustrates a stretch of a representative recording showing the firing pattern of a vagal preganglionic neuron of the NA that was active at rest; (*B*) Time-condensed histograms of the action potential FR illustrating changes in the activity of three vagal preganglionic neurons of the NA during low- and high-intensity simulated exercise, recorded in three separate experiments in sedentary rats. Examples illustrate the responses of one NA neuron with ongoing discharge and the responses of two neurons that were silent at rest and recruited during exercise. Insets illustrate the locations of the recorded cells within the region of the NA. IO, inferior olive; (*C*) Averaged profiles of changes in action potential FR in response to exercise of eight NA neurons that were active at rest and/or activated during exercise and tested at both the low- and high-intensity stimulations.

In experiments conducted in 17 sedentary rats, a total of 51 vagal neurons were recorded in the region of the right NA. Recorded units were identified as vagal preganglionic neurons by antidromic activation in response to electrical stimulation (1.5 mA, 0.1–1 ms pulses, 1 Hz) of the mid-cervical vagus nerve (*Figure 3A*). The mean calculated axonal conduction velocity of NA projections was 5.1 ± 0.6 m/s (range 0.7–16.4 m/s). Five (10%) of the recorded NA neurons had an ongoing activity (range 0.3–10.8 Hz) (*Figure 3A*), while the majority of identified neurons (46 or 90%) did not generate action potentials at rest. These characteristics of the recorded neuronal population are fully consistent with the known properties of cardiac vagal preganglionic neurons of the NA, reported in earlier studies.^15,42^

**Figure 4:**
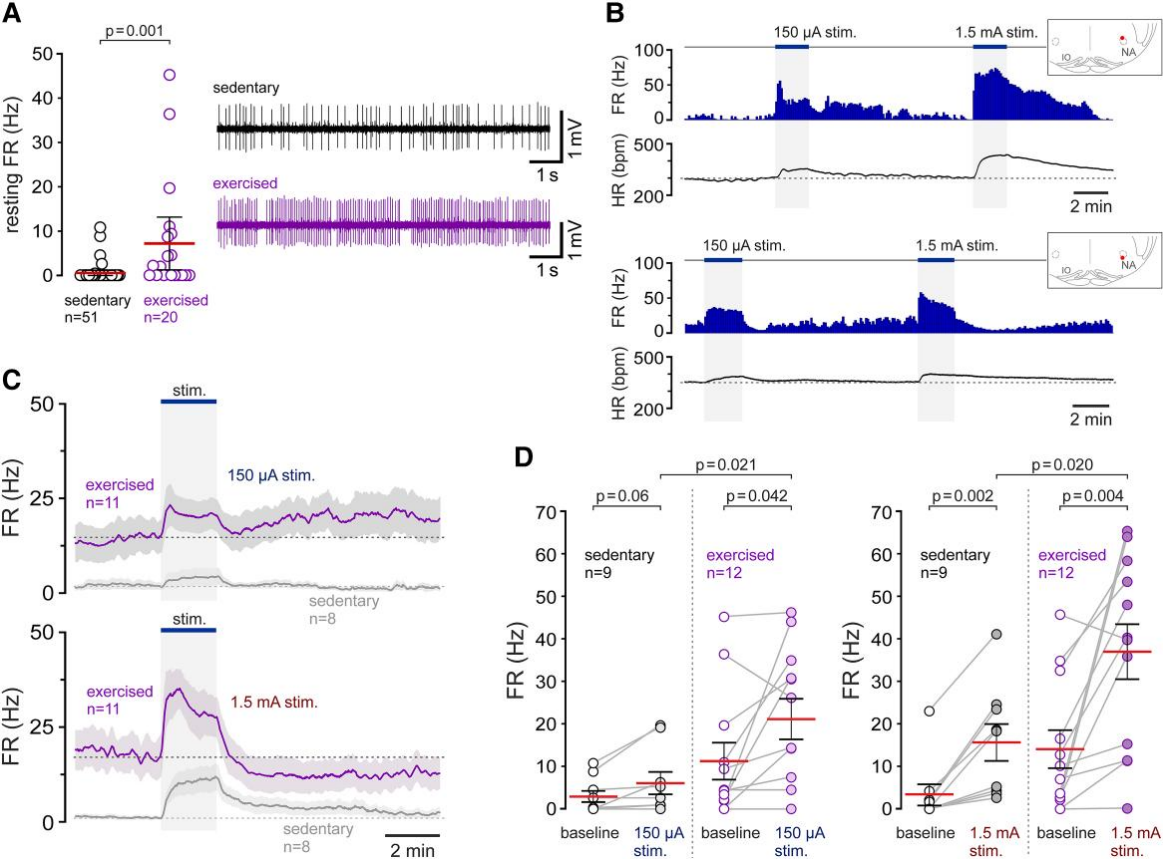
Exercise training increases the resting activity and augments exercise-induced increases in the discharge of vagal preganglionic neurons of the NA. (*A*) Summary data and representative examples of the action potential firing illustrating the resting activity of NA neurons recorded in sedentary and exercise-trained rats. *P* value, Mann–Whitney *U* test; (*B*) Time-condensed records of the action potential FR illustrating two representative examples of the responses of vagal preganglionic neurons of the NA during low- and high-intensity simulated exercise, recorded in two separate experiments conducted in rats that had unrestricted access to running wheels for 6 weeks. Insets illustrate the locations of the recorded cells within the region of the NA. IO, inferior olive; (*C*) Averaged profiles of exercise-induced changes in action potential FR of eleven vagal preganglionic neurons recorded in the NA of exercise-trained rats. Grey traces illustrate the responses of the NA neurons recorded in the experiments in sedentary rats (from *Figure 3C*) and plotted on the same scale for comparison; (*D*) Summary data illustrating the resting FR and peak responses to low- and high-intensity exercise of vagal preganglionic neurons recorded in the NA of sedentary and exercise-trained rats. Only the neurons that were active at rest and/or activated during exercise were included in this analysis. *P* values, ANOVA.

All five NA neurons with ongoing discharge increased their activity during periods of muscular contractions induced by sciatic nerve stimulation. Exercise also recruited the activity of 4 additional neurons that were inactive at rest. Thus, 9 out of 51 (∼18%) of the recorded NA neurons increased their activity in response to exercise. *Figure 3B, C* illustrates the individual examples and the averaged response profiles of NA neurons that were tested at both low- and high-intensity stimulations. Nine NA neurons that were active at rest and/or activated during exercise increased their discharge rate on average from 3.0 ± 1.4 Hz to 6.1 ± 2.6 Hz (*P* = 0.06) and from 3.5 ± 2.5 Hz to 15.8 ± 4.3 Hz (*P* = 0.002) in response to low- and high-intensity exercise, respectively (*Figure 4D*).

### 3.3 Higher resting activity and augmented exercise-induced increases in discharge of vagal preganglionic neurons of the NA after exercise training

Electrophysiological recordings were made from 20 NA neurons in a separate group of six rats that were housed in standard home cages, with unrestricted access to running wheels for 6 weeks (voluntary exercise training). The recorded vagal neurons in this sample had a mean axonal conduction velocity of 5.4 ± 0.9 m/s (range 0.7–14.0 m/s), similar (*P* = 0.8) to that recorded in the NA of sedentary rats.

Exercise training increased the resting action potential firing rate (FR) and the proportion of NA neurons that were active at rest (*P* = 0.001; *Figure 4A*). Ten neurons (50%) of the recorded sample displayed a high level of ongoing discharge (range 2.2–45.2 Hz), and 10 of the recorded units were silent. The majority (9 out of 10) of NA neurons with ongoing discharge were strongly activated during bouts of muscular contractions induced by sciatic nerve stimulation (*Figure 4B, C*). One active neuron was inhibited. Simulated exercise recruited the activity of three additional neurons that had no spontaneous activity at rest. Recording quality of one active neuron deteriorated during data capture and the protocol was not completed. Twelve neurons recorded in the NA of exercise-trained animals which were active at rest and/or activated during exercise increased their mean discharge rate from 11.2 ± 4.4 Hz to 21.1 ± 4.8 Hz (*P* = 0.042) and from 14.0 ± 4.5 Hz to 36.9 ± 6.5 Hz (*P* = 0.004) in response to low- and high-intensity stimulation, respectively (*Figure 4D*). Thus, in exercise-trained rats, the resting activity and peak exercise-induced increases in discharge of NA vagal preganglionic neurons were significantly higher compared with those of sedentary animals.

### 3.4 Exercise increases the activity of vagal preganglionic neurons of the DVMN

**Figure 5:**
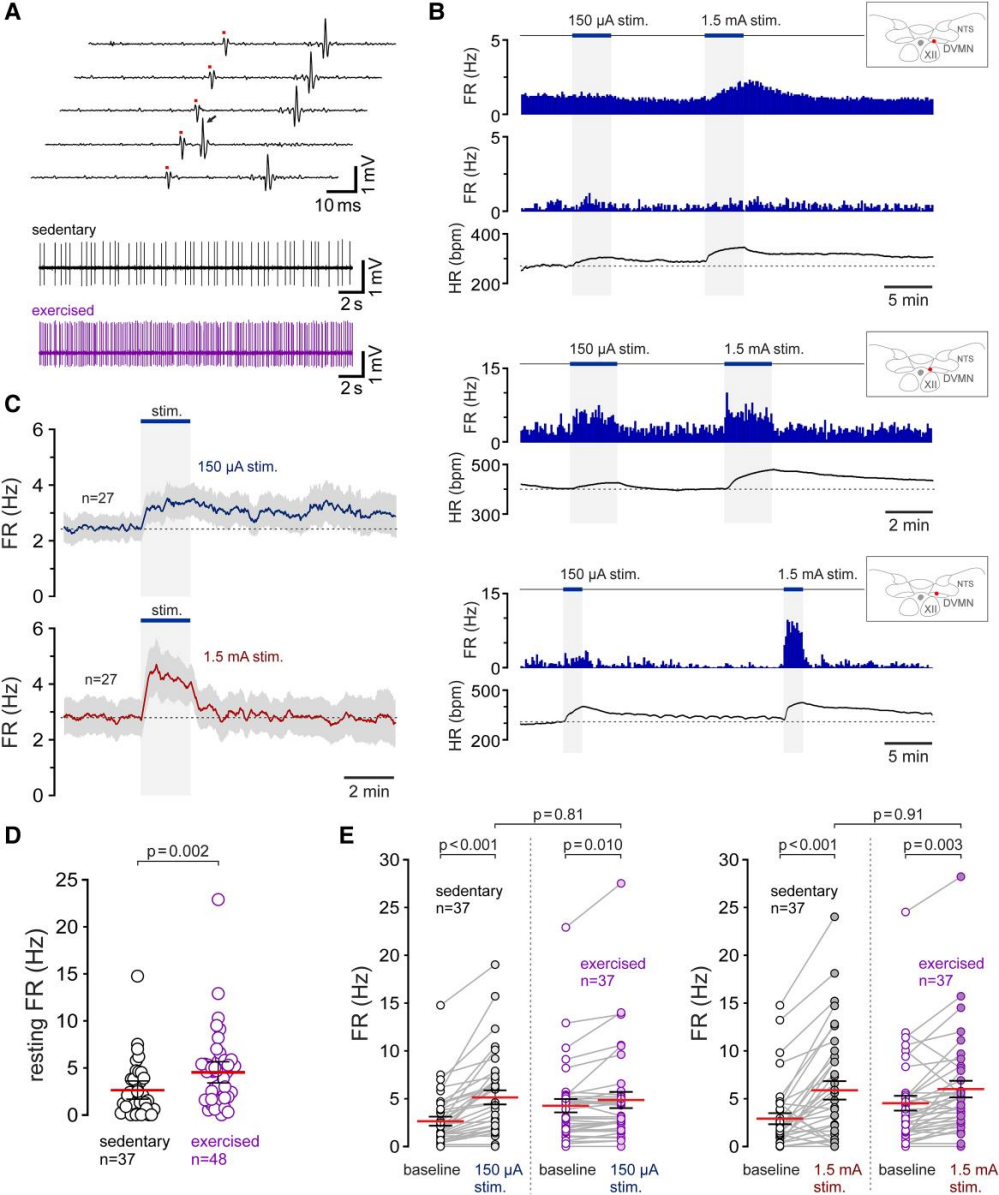
Exercise and exercise training increase the activity of vagal preganglionic neurons of the DVMN. (*A*) Identification of vagal neurons of the DVMN. Five traces illustrate antidromic activation (latency 32 ms) of a vagal preganglionic neuron of the DVMN in response to pulses of electrical stimulation (1.5 mA, 1 ms pulses, 1 Hz) applied to the cervical vagus nerve at the time points marked by the dots. One of the traces shows that a spontaneous spike (arrow) cancels the antidromic spike. Lower traces illustrate the representative examples of the action potential firing of DVMN vagal preganglionic neurons recorded in sedentary and exercise-trained rats; (*B*) Time-condensed histograms of the action potential FR illustrating changes in the activity of four vagal preganglionic neurons of the DVMN responding to low- and high-intensity simulated exercise, recorded in three separate experiments conducted in sedentary rats. Insets illustrate the locations of the recorded cells in the DVMN. XII, hypoglossal nucleus. NTS, nucleus of the solitary tract; (*C*) Averaged profiles of exercise-induced changes in the action potential FR of 27 DVMN neurons that were tested at both low- and high-intensity stimulations in sedentary rats; (*D*) Summary data illustrating the resting action potential FR of all DVMN neurons recorded in sedentary and exercise-trained rats. *P* value, Mann–Whitney *U* test; (*E*) Summary data illustrating the responses of the DVMN vagal preganglionic neurons to low- and high-intensity exercise recorded in the experiments conducted in sedentary and exercise-trained rats. *P* values, ANOVA.

Thirty-seven neurons were recorded in the caudal regions of the DVMN in the experiments conducted in 21 rats. Recorded units were identified as vagal preganglionic neurons by antidromic activation in response to pulses of electrical stimulation applied to the cervical vagus nerve (*Figure 5A*). The calculated mean axonal conduction velocity of recorded DVMN neurons was 1.0 ± 0.2 m/s (range 0.5–4.7 m/s). The majority of recorded DVMN neurons (32 or 86%) had ongoing activity (range 0.2–14.7 Hz) (*Figure 5A*); five neurons in this sample did not generate action potentials at rest. The properties of the recorded neuronal population are consistent with the characteristics of DVMN vagal preganglionic neurons, which in rats have slowly conducting non-myelinated (C fibre) axons.^43^

Individual examples and the averaged response profiles of DVMN vagal preganglionic neurons that were tested at both low- and high-intensity stimulations are illustrated in *Figure 5B, C*. During low-intensity exercise, DVMN neurons increased their discharge rate on average from 2.6 ± 0.5 Hz to 5.1 ± 0.7 Hz (*P* < 0.001) (*Figure 5E*). Low-intensity stimulations did not recruit the activity of 5 neurons that were inactive at rest, and none of the recorded cells was inhibited. In response to high-intensity stimulation, 27 of the 37 recorded DVMN neurons (73%) were strongly activated, and five neurons were inhibited. On average, the population of DVMN vagal neurons sampled in these experiments increased their discharge rate during high-intensity stimulation from 2.9 ± 0.6 Hz to 5.9 ± 1.0 Hz (*P* < 0.001) (*Figure 5E*).

### 3.5 Exercise training increases the resting activity of vagal preganglionic neurons of the DVMN

Forty-eight DVMN vagal preganglionic neurons were recorded in the experiments conducted in 14 rats that had unrestricted access to running wheels for 6 weeks (voluntary exercise training). The recorded neurons in this sample had a mean axonal conduction velocity of 1.0 ± 0.1 m/s (range 0.5–5.0 m/s), similar (*P* = 0.99) to that recorded in the DVMN of sedentary rats. The majority of neurons recorded in the DVMN of exercised rats (47 or 98%) displayed significant ongoing activity (range 0.3–22.9 Hz) (*Figure 5A, D*); only one neuron in this sample did not generate action potentials at rest. The mean resting FR of vagal neurons recorded in the DVMN of exercised rats was significantly (by 73%) higher than the mean FR of DVMN neurons recorded in sedentary rats (4.5 ± 0.6 Hz vs. 2.6 ± 0.5 Hz; *P* = 0.002; *Figure 5D*).

Stable continuous recordings of action potential firing at baseline and during bouts of both low- and high-intensity exercise were obtained from 37 DVMN neurons of rats subjected to exercise training. During low-intensity stimulation, vagal neurons recorded in the DVMN of exercised rats increased their discharge rate from 4.3 ± 0.7 Hz to 4.9 ± 0.8 Hz (*P* = 0.010) (*Figure 5E*). In response to high-intensity stimulation, DVMN vagal neurons sampled in these experiments increased their discharge rate from 4.5 ± 0.8 Hz to 6.0 ± 0.9 Hz (*P* = 0.003) (*Figure 5E*). These data show that exercise training increases the activity of DVMN vagal preganglionic neurons at rest but does not augment the peak increases in FR of these neurons during bouts of acute exercise.

### 3.6 Exercise increases the activity of the cardiac branch of the vagus nerve

**Figure 6:**
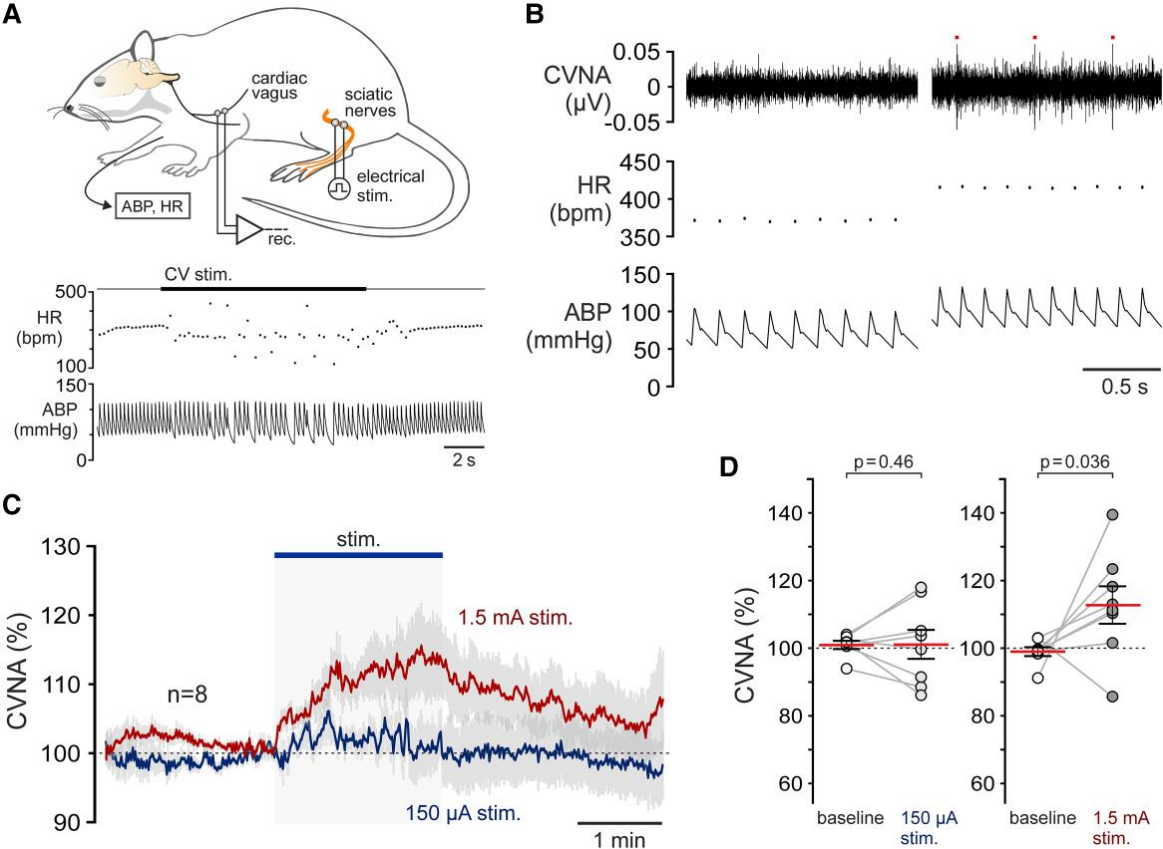
Exercise increases the cardiac vagal nerve activity (CVNA). (*A*) A diagram of the experimental setup in anaesthetized rats instrumented for the recordings of the activity of the cardiac branch of the vagus nerve, ABP, and HR (illustrated on this figure as instantaneous frequency). Electrical stimulation of sciatic nerves was applied to mimic bouts of acute exercise. Representative recordings of HR and ABP responses to electrical stimulation (1 mA, 1 ms pulses, 10 Hz) of the CV are shown; (*B*) Stretches of representative recordings illustrating increases in CVNA in response to high-intensity sciatic nerve stimulation. Three dots above the recording mark stimulus artefacts; (*C*) Averaged activity profiles illustrating changes in CVNA in response to low- and high-intensity exercise; (*D*) Summary data illustrating relative changes in CVNA in response to low- and high-intensity exercise. *P* values, paired *t-*test.

The neuronal recordings described above were sampled from the regions of the NA and DVMN where the cardiac vagal preganglionic neurons reside. The properties of the identified units were fully consistent with the known properties of cardiac vagal neurons reported in earlier studies,^15,42,43^ however, the recorded vagal neurons were not definitively identified as cardiac-projecting. To determine whether the observed exercise-induced responses of individual vagal preganglionic neurons reflect changes in cardiac vagal activity, we performed electrophysiological recordings from the cardiac branch of the vagus nerve in experiments conducted in 15 sedentary rats (*Figure 6A–C*). Stable recordings of the cardiac branch activity during bouts of low- and high-intensity simulated exercise were achieved in eight rats. It was found that high-intensity exercise is associated with significant increases in the activity of the cardiac branch of the vagus nerve (by 13.7 ± 6.6%; *P* = 0.036; *n* = 8), but low-intensity stimulation had no effect (*P* = 0.45; *n* = 8) (*Figure 6D, E*).

### 3.7 Optogenetic stimulation of DVMN neurons modulates gene expression in the SA node

Vagal preganglionic neurons of the NA have a major restraining influence on cardiac nodal tissue, with bradycardia reported in response to stimulation of a single NA neuron.^15^ In contrast, strong activation of the entire DVMN neuronal population produces only small changes in HR^34^ and the significance of DVMN cardiac projections in the regulation of HR remains unclear. This last experiment of the study was designed to explore the potential functional significance of exercise-induced increases in the activity of DVMN neurons. There is evidence that DVMN neurons can regulate ventricular responsiveness to sympathetic stimulation via modulation of the myocardial expression of GRK2 and β-arrestin-2;^19^—key proteins that control β-adrenoceptor signalling and desensitization.^44^ We next tested the hypothesis that DVMN neuronal activity can also modulate gene expression in cardiac nodal tissue.

**Figure 7:**
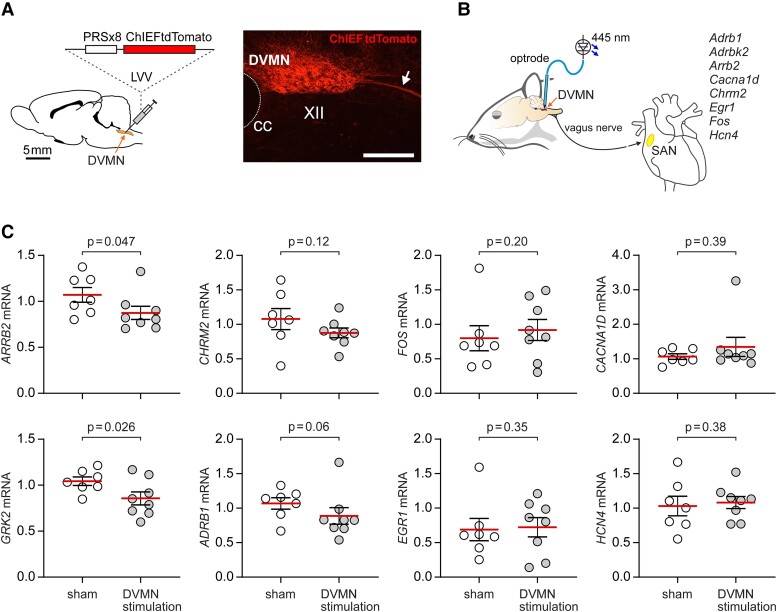
Optogenetic stimulation of the DVMN neurons modulates gene expression in the SA node. (*A*) The DVMN neurons were targeted with a lentiviral vector (LVV) to express light-sensitive protein ChIEF fused with tdTomato under the control of the PRSx8 promoter. A photomicrograph of a representative coronal section of the rat dorsal brainstem illustrates an example of ChIEF-tdTomato expression in the caudal region of the DVMN (Bregma level: −13.8 mm). CC, central canal. XII, hypoglossal motor nucleus. Scale bar: 200 μm. Arrow points to the axons of the transduced DVMN neurons; (*B*) Schematic drawing of the experimental design in anaesthetized rats instrumented for the stimulation of the DVMN neurons expressing ChIEF-tdTomato by application of 445 nm laser light followed by the analysis of the expression of the select genes in the sinoatrial node (SAN); (*C*) Summary data illustrating the effect of light stimulation of the DVMN neurons or sham-stimulation (light delivery in animals transduced to express eGFP in the DVMN) on the relative expression of genes encoding proteins of the G-protein-coupled pathways (β1-adrenoceptors, M2 receptors, GRK2, β-Arrestin-2), key ion channels (HCN4, Ca_v_1.3), and immediate-early genes (C-Fos, early growth response 1) in the SA node. *P* values, Mann–Whitney *U* test.

DVMN neurons were transduced to express light-sensitive channel ChIEF (*Figure 7A*) and stimulated for 4 h with pulses of light, delivered at 5 Hz (to mimic increases in DVMN discharge recorded during acute exercise and after exercise training *Figure 5D, E*). The expression of genes in the SA node encoding proteins of the relevant G-protein-coupled pathways (β1-adrenoceptors, M2 receptors, GRK2, β-Arrestin-2), key ion channels involved in pacemaking and diastolic depolarisation (HCN4, Cav1.3—the α1d subunit of the L-type Ca^2+^ channel) and immediate-early genes (c-Fos, Early Growth Response 1) was analysed (*Figure 7B*). It was found that stimulation of the DVMN neurons resulted in downregulation of β-arrestin-2 and GRK2 mRNA in the SA node (*Figure 7C*). The expression of other genes studied in this experiment was not affected by the stimulation of DVMN neurons.

## 4. Discussion

In this study, we performed microelectrode recordings of vagal preganglionic neuron activity to characterize the immediate and long-term changes in vagal parasympathetic drive in response to exercise and exercise training. Direct recordings of single-unit neuronal action potential firing in the NA and the DVMN demonstrated that both populations of vagal preganglionic neurons are strongly activated during exercise, although neurons of the NA and the DVMN are different in their discharge pattern, cellular properties, and functional role.^14^ The obtained data also showed that exercise training increases the resting activity of both populations of vagal neurons and augments the excitatory responses of the NA neurons during exercise.

### 4.1 On the rapid vagal withdrawal and autonomic control of the heart during exercise

The conventional view of autonomic control of the heart during exercise has long held that increases in HR from resting baseline occur through vagal withdrawal (complete withdrawal at heart rates above 100 bpm in humans), followed by gradual activation of sympathetic input. The idea of vagal withdrawal stems from the original study by Robinson *et al.*,^45^ involving pharmacological autonomic blockade in subjects performing supine cycling exercise. Later studies provided experimental evidence of rapid baroreflex re-setting during exercise (see Refs. ^11,12,46,47^). More recently the field has been reappraised in two comprehensive reviews.^4,5^ Mathematical modelling and analysis of results obtained in studies with autonomic blockade and baroreflex stimulation during exercise in healthy human volunteers suggested that significant cardiac vagal activity could be maintained throughout the exercise.^4^ However, there is only indirect evidence supporting these ideas as the dynamic changes in vagal activity during exercise with increasing workload have never been directly assessed. HRV analysis was used in some studies to evaluate the strength of vagal modulation of HR during exercise (reviewed in Ref. ^5^), however, HRV metrics cannot be used with certainty to measure autonomic activity.^7,8,48^

In this study, we used an experimental model of simulated exercise in anaesthetized animals. The model involves low-frequency stimulation of sciatic nerves to activate hindlimb motor and sensory nerves to produce rhythmic hindlimb muscular contractions, increase plasma concentration of lactate, and mimic the feedback from muscle mechanoreceptors, resulting in intensity-dependent simultaneous increases in HR and ABP (baroreceptor resetting^4^), similar to these occurring during ‘natural’ exercise. Importantly, the effects of muscarinic and/or β-adrenoceptor blockade on the cardiovascular responses evoked in this model are very similar to those observed in human exercise studies with autonomic blockade (see e.g. Ref. ^41^).

Both populations of vagal preganglionic neurons in the NA and DVMN were found to increase their activity during exercise. Considering that neurons of the NA primarily control the nodal tissue and, therefore, HR, and in accord with the prevailing model of autonomic control of the heart during exercise, immediate inhibition of NA activity in response to exercise was expected. In total 71 neurons were recorded from the region of the NA where the cardiac vagal preganglionic neurons reside; out of 15 neurons with ongoing discharge, only one neuron was inhibited, whilst the rest were strongly activated during exercise. Moreover, exercise recruited the activity of seven additional NA neurons that had no resting activity. Although the recorded neurons were not definitively identified as cardiac-projecting, recordings from the cardiac vagal branch showed that the activity of the CV increases during exercise, consistent with the results obtained in the experiments involving recordings of individual vagal preganglionic neurons. The response profiles of vagal neuron firing were found to be remarkably similar to the profiles of HR changes induced in this exercise model (compare *Figures 2B* and *3C*). Thus, during exercise, central vagal activity increases and decreases in parallel with and in the same direction as HR changes.

It could be argued that the experimental model used in this study lacks a key component that contributes to the development of the cardiovascular response during exercise, namely the so-called ‘central command’, collectively representing inputs from higher brain centres.^38^ Kadowaki and colleagues^49^ recorded the activity of the cardiac branch of the vagus nerve during fictive motor activity that occurred spontaneously in unanesthetized, decerebrate cats. Similar to the results of the present study, the authors found that the activity of the CV never decreases but always increases throughout the ‘central command’-induced motor activity.^49^

The data obtained in this study strongly suggest that cardiovascular responses during exercise are driven primarily by increases in sympathetic nerve activity.^50–52^ We hypothesize that the remaining HR responses to exercise that is observed under conditions of systemic β-adrenoceptor blockade (that led to the idea of vagal withdrawal; see *Figure 2B*) develop as a result of sympathetic activity-mediated presynaptic inhibition of acetylcholine release from the terminals of vagal pre- and/or postganglionic neurons at the level of the cardiac ganglia and/or cardiac nodal tissue.

### 4.2 What is the functional significance of increased vagal activity during exercise?

There is significant evidence obtained in studies using experimental animal models suggesting that simultaneous activation of parasympathetic and sympathetic nerves innervating the heart may occur under various conditions (reviewed in Ref. ^53^). Indeed, the original idea of complete vagal withdrawal during exercise seems rather implausible; although the baroreflex is reset to a higher operating point during exercise, cardiac vagal activity is still required for baroreflex control of the heart at any level of exercise intensity.^11^ Paton and colleagues suggested that simultaneous co-activation of both autonomic limbs leads to more efficient cardiac function and greater cardiac output compared to that resulting from the activation of the sympathetic limb alone.^53^

We previously reported that vagal efferent stimulation increases cardiac contractility and improves exercise tolerance; these effects were found to be associated with reduced expression of GRK2 and β-arrestin-2 in the ventricular myocardium.^19,34,54^ In the experiments designed to explore the potential functional significance of exercise-induced increases in DVMN activity in the regulation of HR, we found that optogenetic stimulation of vagal projections originating from the DVMN has the same effect and reduces the expression of GRK2 and β-arrestin-2 in the SA node. These data support the hypothesis that enhanced vagal activity optimizes HR responses to sympathetic stimulation by inhibiting myocardial expression of the key negative regulators of β-adrenoceptor-mediated signalling. However, the mechanisms responsible for the transcriptional effects of increased vagal activity on the heart remain to be identified.

Increased cardiac vagal activity may also contribute to the regulation of coronary blood flow during exercise. There is evidence that vagus nerve stimulation increases coronary blood flow, and this effect is mediated by the actions of vasoactive intestinal peptide.^55^ However, the functional significance of vagal control of coronary blood flow is not entirely clear as all the supporting data come from studies involving electrical stimulation of the vagus nerve (e.g. see Ref. ^55^). There is also no evidence at present to suggest the existence of a separate pool of vagal preganglionic neurons specifically controlling coronary blood flow. There is evidence that vagal innervation of the ventricles is provided by the DVMN neuronal projections that modulate ventricular excitability and contractility.^13,17–19^ Therefore, it is plausible that activation of the cardiac-projecting DVMN vagal preganglionic neurons may contribute to the increases in coronary blood flow during exercise.

### 4.3 On the mechanisms of exercise training-induced bradycardia

Regular exercise lowers resting HR which in healthy individuals is largely determined by the restraining influence of the vagus nerve on cardiac pacemaker cells.^6^ Therefore, lower heart rates in trained individuals have been traditionally attributed to high parasympathetic vagal tone.^56^ Studies conducted in one of our laboratories also suggested that exercise training-induced bradycardia develops as a result of training-induced electrical remodelling of the SA node resulting in a reduced intrinsic rate of nodal myocytes.^36,37^ In a debate article, D’Souza and colleagues^57^ argued that the notion of high vagal tone being responsible for training-induced lowering of resting HR had never been supported by direct experimental evidence, such as electrophysiological recordings of vagal efferent activity.

This study provides such evidence by showing that voluntary exercise training increases the resting activity of vagal neurons of the NA and DVMN and augments the excitatory responses of the NA neurons during exercise. The data suggest that cardiac vagal activity can indeed be ‘trained’ by exercise. We propose that recurrent increases in the activity of vagal preganglionic neurons during bouts of exercise lead to higher baseline discharge of these neurons, and, therefore, higher resting vagal tone contributing to the lower heart rates that are associated with training. The signalling and cellular mechanisms responsible for autonomic adaptation to regular exercise are a topic for future investigation. The notion of central vagal withdrawal during exercise (which would require inhibition, rather than excitation, of vagal neurons during exercise) appears to be at odds with our understanding of brain plasticity, as repeated stimulations and activations of neurons and networks are required for sustained increases in activity.^58^

### 4.4 Heart rate recovery after exercise

In contrast to HRV metrics, a highly reproducible and robust measure of cardiac vagal activity in humans is the speed of heart rate recovery (HRR) after exercise. After HR peaks during bouts of acute exercise, a variably steep decline occurs upon cessation of exercise, termed HRR. It is generally believed that HRR after exercise is mediated by rapid vagal reactivation,^59^ as the speed of HRR is greatly reduced by systemic muscarinic receptor blockade^40^ (also see *Figure 2B*). The data obtained in this study warrant a reconsideration of this view. We propose that HRR is not a measure of rapid vagal reactivation, but reflects the strength of cardiac vagal tone at the end of the exercise, revealed after withdrawal of sympathetic activity,^51^ which during exercise overrides the vagal restraint via presynaptic inhibition at the level of the cardiac ganglia and/or nodal tissue. Exercise-induced central vagal plasticity, leading to higher resting discharge and augmented responses of vagal preganglionic neurons during exercise, provides evidence in support of this hypothesis. It is also consistent with the data obtained in studies conducted in athletes,^60,61^ patients with heart failure^62–64^ and type 2 diabetes,^65^ all showing that exercise training increases the speed of HRR, indicative of higher cardiac vagal activity at the end of the exercise.

### 4.5 Summary

The data obtained in this study show that central vagal parasympathetic drive increases during exercise and provide the first direct neurophysiological evidence in support of the long-standing hypothesis that exercise training increases vagal tone. The data argue against the notion of exercise-induced central vagal withdrawal during exercise. It is hypothesized that maintained/recruited vagal activity is important for optimizing cardiac responses during exercise. We suggest that robust increases in the activity of vagal preganglionic neurons during bouts of exercise underlie activity-dependent plasticity, leading to higher resting vagal tone, which confers multiple health benefits of regular exercise.

## Data Availability

The data that support the findings of this study are available from the corresponding author upon reasonable request.

## References

[1] Blair SN , Kohl HW III, Paffenbarger RS Jr, Clark DG, Cooper KH, Gibbons LW. Physical fitness and all-cause mortality. A prospective study of healthy men and women. JAMA 1989;262:2395–2401. 2795824 10.1001/jama.262.17.2395

[2] Lee IM , Hsieh CC, Paffenbarger RS Jr. Exercise intensity and longevity in men. The Harvard Alumni Health Study. JAMA 1995;273(15):1179–1184. 7707624

[3] Blair SN , Kohl HW III, Barlow CE, Paffenbarger RS, Gibbons LW, Macera CA. Changes in physical fitness and all-cause mortality. A prospective study of healthy and unhealthy men. JAMA 1995;273(14):1093–1098. 7707596

[4] White DW , Raven PB. Autonomic neural control of heart rate during dynamic exercise: revisited. J Physiol 2014;592(12):2491–2500. 24756637 10.1113/jphysiol.2014.271858 PMC4080933

[5] Michael S , Graham KS, Davis GO. Cardiac autonomic responses during exercise and post-exercise recovery using heart rate variability and systolic time intervals-a review. Front Physiol 2017;8:301. 28611675 10.3389/fphys.2017.00301 PMC5447093

[6] Gourine AV , Ackland GL. Cardiac vagus and exercise. Physiology (Bethesda) 2019;34(1):71–80. 30540229 10.1152/physiol.00041.2018 PMC6383634

[7] Monfredi O , Lyashkov AE, Johnsen AB, Inada S, Schneider H, Wang R, Nirmalan M, Wisloff U, Maltsev VA, Lakatta EG, Zhang H, Boyett MR. Biophysical characterization of the underappreciated and important relationship between heart rate variability and heart rate. Hypertension 2014;64(6):1334–1343. 25225208 10.1161/HYPERTENSIONAHA.114.03782 PMC4326239

[8] Marmerstein JT , McCallum GA, Durand DM. Direct measurement of vagal tone in rats does not show correlation to HRV. Sci Rep 2021;11(1):1210. 33441733 10.1038/s41598-020-79808-8 PMC7807082

[9] O'Leary DS , Seamans DP. Effect of exercise on autonomic mechanisms of baroreflex control of heart rate. J Appl Physiol (1985) 1993;75(5):2251–2257. 7905872 10.1152/jappl.1993.75.5.2251

[10] Ogoh S , Fisher JP, Dawson EA, White MJ, Secher NH, Raven PB. Autonomic nervous system influence on arterial baroreflex control of heart rate during exercise in humans. J Physiol 2005;566(Pt 2):599–611. 15890708 10.1113/jphysiol.2005.084541 PMC1464761

[11] Raven PB , Fadel PJ, Ogoh S. Arterial baroreflex resetting during exercise: a current perspective. Exp Physiol 2006;91(1):37–49. 16210446 10.1113/expphysiol.2005.032250

[12] Ogoh S , Brothers RM, Barnes Q, Eubank WL, Hawkins MN, Purkayastha S, Yurvati A, Raven PB. Cardiopulmonary baroreflex is reset during dynamic exercise. J Appl Physiol (1985) 2006;100(1):51–59. 16150844 10.1152/japplphysiol.00804.2005

[13] Geis GS , Wurster RD. Cardiac responses during stimulation of the dorsal motor nucleus and nucleus ambiguus in the cat. Circ Res 1980;46(5):606–611. 7363410 10.1161/01.res.46.5.606

[14] Gourine AV , Machhada A, Trapp S, Spyer KM. Cardiac vagal preganglionic neurones: an update. Auton Neurosci 2016;199:24–28. 27396874 10.1016/j.autneu.2016.06.003

[15] McAllen RM , Spyer KM. Two types of vagal preganglionic motoneurones projecting to the heart and lungs. J Physiol 1978;282:353–364. 722537 10.1113/jphysiol.1978.sp012468 PMC1282744

[16] Veerakumar A , Yung AR, Liu Y, Krasnow MA. Molecularly defined circuits for cardiovascular and cardiopulmonary control. Nature 2022;606(7915):739–746. 35650438 10.1038/s41586-022-04760-8 PMC9297035

[17] Machhada A , Ang R, Ackland GL, Ninkina N, Buchman VL, Lythgoe MF, Trapp S, Tinker A, Marina N, Gourine AV. Control of ventricular excitability by neurons of the dorsal motor nucleus of the vagus nerve. Heart Rhythm 2015;12(11):2285–2293. 26051529 10.1016/j.hrthm.2015.06.005 PMC4631809

[18] Machhada A , Marina N, Korsak A, Stuckey DJ, Lythgoe MF, Gourine AV. Origins of the vagal drive controlling left ventricular contractility. J Physiol 2016;594(14):4017–4030. 26940639 10.1113/JP270984 PMC4945717

[19] Machhada A , Trapp S, Marina N, Stephens RCM, Whittle J, Lythgoe MF, Kasparov S, Ackland GL, Gourine AV. Vagal determinants of exercise capacity. Nat Commun 2017;8:15097. 28516907 10.1038/ncomms15097 PMC5454375

[20] Dale N , Gourine AV, Llaudet E, Bulmer D, Thomas T, Spyer KM. Rapid adenosine release in the nucleus tractus solitarii during defence response in rats: real-time measurement in vivo. J Physiol 2002;544(Pt 1):149–160. 12356888 10.1113/jphysiol.2002.024158 PMC2290567

[21] Gourine AV , Atkinson L, Deuchars J, Spyer KM. Purinergic signalling in the medullary mechanisms of respiratory control in the rat: respiratory neurones express the P2X_2_ receptor subunit. J Physiol 2003;552(Pt 1):197–211. 12878756 10.1113/jphysiol.2003.045294 PMC2343330

[22] Gourine AV , Dale N, Korsak A, Llaudet E, Tian F, Huckstepp R, Spyer KM. Release of ATP and glutamate in the nucleus tractus solitarii mediate pulmonary stretch receptor (Breuer-Hering) reflex pathway. J Physiol 2008;586(16):3963–3978. 18617567 10.1113/jphysiol.2008.154567 PMC2538935

[23] Mastitskaya S , Turovsky E, Marina N, Theparambil SM, Hadjihambi A, Kasparov S, Teschemacher AG, Ramage AG, Gourine AV, Hosford PS. Astrocytes modulate baroreflex sensitivity at the level of the nucleus of the solitary tract. J Neurosci 2020;40(15):3052–3062. 32132265 10.1523/JNEUROSCI.1438-19.2020 PMC7141885

[24] Hosford PS , Wells JA, Christie IN, Lythgoe MF, Millar J, Gourine AV. Electrochemical carbon fiber-based technique for simultaneous recordings of brain tissue PO_2_, pH, and extracellular field potentials. Biosens Bioelectron X 2019;3:100034. 10.1016/j.biosx.2020.100034 PMC7357830 32685919

[25] Waldrop TG , Mullins DC, Henderson MC. Effects of hypothalamic lesions on the cardiorespiratory responses to muscular contraction. Respir Physiol 1986;66(2):215–224. 3809757 10.1016/0034-5687(86)90074-5

[26] Kramer JM , Aragones A, Waldrop TG. Reflex cardiovascular responses originating in exercising muscles of mice. J Appl Physiol (1985) 2001;90(2):579–585. 11160056 10.1152/jappl.2001.90.2.579

[27] Kaufman MP , Rybicki KJ, Waldrop TG, Mitchell JH. Effect on arterial pressure of rhythmically contracting the hindlimb muscles of cats. J Appl Physiol Respir Environ Exerc Physiol 1984;56(5):1265–1271. 6327583 10.1152/jappl.1984.56.5.1265

[28] Waldrop TG , Mullins DC, Millhorn DE. Control of respiration by the hypothalamus and by feedback from contracting muscles in cats. Respir Physiol 1986;64(3):317–328. 3738257 10.1016/0034-5687(86)90125-8

[29] Korsak A , Sheikhbahaei S, Machhada A, Gourine AV, Huckstepp RTR. The role of parafacial neurons in the control of breathing during exercise. Sci Rep 2018;8(1):400. 29321559 10.1038/s41598-017-17412-z PMC5762684

[30] Narath E , Skalicky M, Viidik A. Voluntary and forced exercise influence the survival and body composition of ageing male rats differently. Exp Gerontol 2001;36(10):1699–1711. 11672990 10.1016/s0531-5565(01)00145-0

[31] Judge S , Jang YM, Smith A, Selman C, Phillips T, Speakman JR, Hagen T, Leeuwenburgh C. Exercise by lifelong voluntary wheel running reduces subsarcolemmal and interfibrillar mitochondrial hydrogen peroxide production in the heart. Am J Physiol Regul Integr Comp Physiol 2005;289(6):R1564–R1572. 16051717 10.1152/ajpregu.00396.2005

[32] Lonergan T , Teschemacher AG, Hwang D-Y, Kim K-S, Kasparov S. Targeting brainstem centres of cardiovascular control using adenoviral vectors: impact of promoters on transgene expression. Physiol Genomics 2005;20(2):165–172. 15561757 10.1152/physiolgenomics.00120.2004

[33] Mastitskaya S , Marina N, Gourine A, Gilbey MP, Spyer KM, Teschemacher AG, Kasparov S, Trapp S, Ackland GL, Gourine AV. Cardioprotection evoked by remote ischaemic preconditioning is critically dependent on the activity of vagal pre-ganglionic neurones. Cardiovasc Res 2012;95(4):487–494. 22739118 10.1093/cvr/cvs212 PMC3422080

[34] Machhada A , Hosford PS, Dyson A, Ackland GL, Mastitskaya S, Gourine AV. Optogenetic stimulation of vagal efferent activity preserves left ventricular function in experimental heart failure. JACC Basic Transl Sci 2020;5(8):799–810. 32875170 10.1016/j.jacbts.2020.06.002 PMC7452237

[35] Booth LC , Yao ST, Korsak A, Farmer DGS, Hood SG, McCormick D, Boesley Q, Connelly AA, McDougall SJ, Korim WS, Guild SJ, Mastitskaya S, Le P, Teschemacher AG, Kasparov S, Ackland GL, Malpas SC, McAllen RM, Allen AM, May CN, Gourine AV. Selective optogenetic stimulation of efferent fibers in the vagus nerve of a large mammal. Brain Stimul 2021;14(1):88–96. 33217609 10.1016/j.brs.2020.11.010 PMC7836098

[36] D'Souza A , Bucchi A, Johnsen AB, Logantha SJ, Monfredi O, Yanni J, Prehar S, Hart G, Cartwright E, Wisloff U, Dobryznski H, DiFrancesco D, Morris GM, Boyett MR. Exercise training reduces resting heart rate via downregulation of the funny channel HCN4. Nat Commun 2014;5:3775. 24825544 10.1038/ncomms4775 PMC4024745

[37] D'Souza A , Pearman CM, Wang Y, Nakao S, Logantha SJRJ, Cox C, Bennett H, Zhang Y, Johnsen AB, Linscheid N, Poulsen PC, Elliott J, Coulson J, McPhee J, Robertson A, da Costa Martins PA, Kitmitto A, Wisloff U, Cartwright EJ, Monfredi O, Lundby A, Dobrzynski H, Oceandy D, Morris GM, Boyett MR. Targeting miR-423-5p reverses exercise training-induced HCN4 channel remodeling and sinus bradycardia. Circ Res 2017;121(9):1058–1068. 28821541 10.1161/CIRCRESAHA.117.311607 PMC5636198

[38] Nobrega AC , O'Leary D, Silva BM, Marongiu E, Piepoli MF, Crisafulli A. Neural regulation of cardiovascular response to exercise: role of central command and peripheral afferents. Biomed Res Int 2014;2014:478965. 10.1155/2014/478965 PMC4000959 24818143

[39] O'Leary DM , Jones JF. Discharge patterns of preganglionic neurones with axons in a cardiac vagal branch in the rat. Exp Physiol 2003;88(6):711–723. 14603369 10.1113/eph8802590

[40] Fisher JP , Seifert T, Hartwich D, Young CN, Secher NH, Fadel PJ. Autonomic control of heart rate by metabolically sensitive skeletal muscle afferents in humans. J Physiol 2010;588(Pt 7):1117–1127. 20142272 10.1113/jphysiol.2009.185470 PMC2852999

[41] Imai K , Sato H, Hori M, Kusuoka H, Ozaki H, Yokoyama H, Takeda H, Inoue M, Kamada T. Vagally mediated heart rate recovery after exercise is accelerated in athletes but blunted in patients with chronic heart failure. J Am Coll Cardiol 1994;24(6):1529–1535. 7930286 10.1016/0735-1097(94)90150-3

[42] McAllen RM , Spyer KM. The location of cardiac vagal preganglionic motoneurones in the medulla of the cat. J Physiol 1976;258(1):187–204. 940054 10.1113/jphysiol.1976.sp011414 PMC1308967

[43] Jones JF , Wang Y, Jordan D. Activity of C fibre cardiac vagal efferents in anaesthetized cats and rats. J Physiol 1998;507(Pt 3):869–880. 9508846 10.1111/j.1469-7793.1998.869bs.x PMC2230810

[44] Kohout TA , Lefkowitz RJ. Regulation of G protein-coupled receptor kinases and arrestins during receptor desensitization. Mol Pharmacol 2003;63(1):9–18. 12488531 10.1124/mol.63.1.9

[45] Robinson BF , Epstein SE, Beiser GD, Braunwald E. Control of heart rate by the autonomic nervous system. Studies in man on the interrelation between baroreceptor mechanisms and exercise. Circ Res 1966;19(2):400–411. 5914852 10.1161/01.res.19.2.400

[46] Rowell LB , O'Leary DS. Reflex control of the circulation during exercise: chemoreflexes and mechanoreflexes. J Appl Physiol (1985) 1990;69(2):407–418. 2228848 10.1152/jappl.1990.69.2.407

[47] Dicarlo SE , Bishop VS. Onset of exercise shifts operating point of arterial baroreflex to higher pressures. Am J Physiol 1992;262(1 Pt 2):H303–H307. 1733319 10.1152/ajpheart.1992.262.1.H303

[48] Hayano J , Yuda E. Pitfalls of assessment of autonomic function by heart rate variability. J Physiol Anthropol 2019;38(1):3. 30867063 10.1186/s40101-019-0193-2 PMC6416928

[49] Kadowaki A , Matsukawa K, Wakasugi R, Nakamoto T, Liang N. Central command does not decrease cardiac parasympathetic efferent nerve activity during spontaneous fictive motor activity in decerebrate cats. Am J Physiol Heart Circ Physiol 2011;300(4):H1373–H1385. 21297027 10.1152/ajpheart.01296.2010

[50] Saito M , Tsukanaka A, Yanagihara D, Mano T. Muscle sympathetic nerve responses to graded leg cycling. J Appl Physiol (1985) 1993;75(2):663–667. 8226466 10.1152/jappl.1993.75.2.663

[51] Tsuchimochi H , Matsukawa K, Komine H, Murata J. Direct measurement of cardiac sympathetic efferent nerve activity during dynamic exercise. Am J Physiol Heart Circ Physiol 2002;283(5):H1896–H1906. 12384467 10.1152/ajpheart.00112.2002

[52] Ichinose M , Saito M, Fujii N, Ogawa T, Hayashi K, Kondo N, Nishiyasu T. Modulation of the control of muscle sympathetic nerve activity during incremental leg cycling. J Physiol 2008;586(11):2753–2766. 18403425 10.1113/jphysiol.2007.150060 PMC2536590

[53] Paton JF , Boscan P, Pickering AE, Nalivaiko E. The yin and yang of cardiac autonomic control: vago-sympathetic interactions revisited. Brain Res Brain Res Rev 2005;49(3):555–565. 16269319 10.1016/j.brainresrev.2005.02.005

[54] Ackland GL , Whittle J, Toner A, Machhada A, Del Arroyo AG, Sciuso A, Jenkins N, Dyson A, Struthers R, Sneyd JR, Minto G, Singer M, Shah AM, Gourine AV. Molecular mechanisms linking autonomic dysfunction and impaired cardiac contractility in critical illness. Crit Care Med 2016;44(8):e614–e624. 26950003 10.1097/CCM.0000000000001606 PMC4950969

[55] Feliciano L , Henning RJ. Vagal nerve stimulation releases vasoactive intestinal peptide which significantly increases coronary artery blood flow. Cardiovasc Res 1998;40(1):45–55. 9876316 10.1016/s0008-6363(98)00122-9

[56] Coote JH , White MJ. Crosstalk proposal: bradycardia in the trained athlete is attributable to high vagal tone. J Physiol 2015;593(8):1745–1747. 25871550 10.1113/jphysiol.2014.284364 PMC4405728

[57] D'Souza A , Sharma S, Boyett MR. Crosstalk opposing view: bradycardia in the trained athlete is attributable to a downregulation of a pacemaker channel in the sinus node. J Physiol 2015;593(8):1749–1751. 25871551 10.1113/jphysiol.2014.284356 PMC4405729

[58] Abraham WC . Metaplasticity: tuning synapses and networks for plasticity. Nat Rev Neurosci 2008;9(5):387. 18401345 10.1038/nrn2356

[59] Coote JH . Recovery of heart rate following intense dynamic exercise. Exp Physiol 2010;95(3):431–440. 19837772 10.1113/expphysiol.2009.047548

[60] Daanen HA , Lamberts RP, Kallen VL, Jin A, Van Meeteren NL. A systematic review on heart-rate recovery to monitor changes in training status in athletes. Int J Sports Physiol Perform 2012;7(3):251–260. 22357753 10.1123/ijspp.7.3.251

[61] Mann TN , Webster C, Lamberts RP, Lambert MI. Effect of exercise intensity on post-exercise oxygen consumption and heart rate recovery. Eur J Appl Physiol 2014;114(9):1809–1820. 24878688 10.1007/s00421-014-2907-9

[62] Myers J , Hadley D, Oswald U, Bruner K, Kottman W, Hsu L, Dubach P. Effects of exercise training on heart rate recovery in patients with chronic heart failure. Am Heart J 2007;153(6):1056–1063. 17540210 10.1016/j.ahj.2007.02.038

[63] Pearson MJ , Smart NA. Exercise therapy and autonomic function in heart failure patients: a systematic review and meta-analysis. Heart Fail Rev 2018;23(1):91–108. 29185161 10.1007/s10741-017-9662-z

[64] Streuber SD , Amsterdam EA, Stebbins CL. Heart rate recovery in heart failure patients after a 12-week cardiac rehabilitation program. Am J Cardiol 2006;97(5):694–698. 16490440 10.1016/j.amjcard.2005.09.117

[65] Bhati P , Shenoy S, Hussain ME. Exercise training and cardiac autonomic function in type 2 diabetes mellitus: a systematic review. Diabetes Metab Syndr 2018;12(1):69–78. 28888482 10.1016/j.dsx.2017.08.015

